# Plant–plant interactions change during succession on nurse logs in a northern temperate rainforest

**DOI:** 10.1002/ece3.7786

**Published:** 2021-06-20

**Authors:** Carrie L. Woods, Katy Maleta, Kimmy Ortmann

**Affiliations:** ^1^ Biology Department University of Puget Sound Tacoma WA USA

**Keywords:** determinants of plant community structure, moss, nurse log, Olympic rainforest, plant–plant interactions, Stress Gradient Hypothesis, structural diversity, temperate rainforest

## Abstract

Plant–plant interactions change through succession from facilitative to competitive. At early stages of succession, early‐colonizing plants can increase the survival and reproductive output of other plants by ameliorating disturbance and stressful conditions. At later stages of succession, plant interactions are more competitive as plants put more energy toward growth and reproduction. In northern temperate rainforests, gap dynamics result in tree falls that facilitate tree regeneration (nurse logs) and bryophyte succession. How bryophyte‐tree seedling interactions vary through log succession remains unclear. We examined the relationships of tree seedlings, bryophyte community composition, bryophyte depth, and percent canopy cover in 166 1.0 m^2^ plots on nurse logs and the forest floor in the Hoh rainforest in Washington, USA, to test the hypothesis that bryophyte‐tree seedling interactions change from facilitative to competitive as the log decays. Tree seedling density was highest on young logs with early‐colonizing bryophyte species (e.g., *Rhizomnium glabrescens*) and lowest on decayed logs with *Hylocomium splendens*, a long‐lived moss that reaches depths >20 cm. As a result, bryophyte depth increased with nurse log decay and was negatively associated with tree seedling density. Tree seedling density was 4.6× higher on nurse logs than on the forest floor, which was likely due to competitive exclusion by forest floor plants, such as *H. splendens*. Nurse logs had 17 species of bryophytes while the forest floor had six, indicating that nurse logs contribute to maintaining bryophyte diversity. Nurse logs enable both tree seedlings and smaller bryophyte species to avoid competition with forest floor plants, including the dominant bryophyte, *H. splendens*. *H. splendens* is likely a widespread driver of plant community structure given its dominance in northern temperate forests. Our findings indicate that plant–plant interactions shift with succession on nurse logs from facilitative to competitive and, thus, influence forest community structure and dynamics.

## Graphical abstract

Our findings corroborate previous research on the importance of plant–plant interactions in community structure and dynamics, and show how these interactions change during succession. While often overlooked, bryophytes played a key role in determining gap‐dynamics forest regeneration.

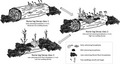

## 1 INTRODUCTION

Plant–plant interactions are complex and can vary from facilitative to competitive depending on the severity of the external environment (Bertness & Callaway, 1994; Brooker & Callaghan, 1998; Callaway & Walker, 1997). Most positive plant interactions have been found to occur in environments that are either considered stressful for plants or have high degrees of disturbance (e.g., arid, salt marshes, polar tundra), and happen through the amelioration of those stressful conditions by the physical presence of another plant (Brooker & Callaghan, 1998; Callaway et al., 2002). In other words, the physical presence of one plant is beneficial to another plant through feedback on the external environment rather than through direct connections between individuals (Brooker & Callaghan, 1998). Facilitation, in particular, appears to be important in colonization and early community development (Callaway & Walker, 1997; Clements, 1916; Connell & Slatyer, 1977; Egler, 1954; Gómez‐Aparicio, 2009). The importance of facilitation in early stages of succession is driven either by intraspecific positive density dependence or interspecific interactions between stress‐tolerant and stress‐intolerant plants (Brooker & Callaghan, 1998). As the community develops, the amelioration of disturbance and stress by the physical presence of plants (e.g., stabilizing slopes, herbivory protection, microclimate, providing substrate, nutrients, and water) results in competitive interactions becoming more important over time as plants can put more of their energy toward growth and reproduction (Brooker & Callaghan, 1998; Egler, 1954; Ricklefs, 1977; Walker & Chapin, 1987; but see Maestre et al., 2005). While vascular plants have largely been the focus of plant–plant interaction research, bryophytes can have facilitative or competitive interactions with vascular plants depending on multiple abiotic and biotic factors (Doxford et al., 2013; Gornall et al., 2011; Gough, 2006; Rehm et al., 2019; Sedia & Ehrenfeld, 2003; Soudzilovskaia et al., 2011; Staunch et al., 2012). In locations where bryophytes dominate the ground cover, such as in the arctic and boreal forest, bryophytes can structure the composition of vascular plant communities as they are often more tolerant of the stressful conditions than the vascular plants that grow among them (Gavini et al., 2019; Gornall et al., 2011; Gough, 2006). In fact, the recovery of native tree species could depend on the recovery of bryophytes in restored habitats. In restoration corridors in Hawaii, native tree seedlings were strongly associated with bryophyte mats, both of which were in low abundance in restoration corridors (Rehm et al., 2019). However, bryophytes are generally treated as a static community, and how various stages of bryophyte succession influence vascular plants remains largely unexplored.

In old‐growth forests, forest regeneration is often triggered by disturbances, such as tree falls, that create gaps into which early‐colonizing species proliferate (i.e., small‐scale gap dynamics, McCarthy, 2001; Ricklefs, 1977; Yamamoto, 2000). These gaps often result in a change in light levels that facilitate regeneration of early‐colonizing shade‐intolerant plants if the gaps are large, and shade‐tolerant climax species if the gaps are small (<200 m^2^); colonization of small gaps by the canopy dominants can perpetuate the current canopy species composition (Denslow et al., 1990; McCarthy, 2001; Runkle, 1981). In Appalachian forests in Tennessee, for example, shade‐intolerant trees were only able to establish in gaps created by multiple tree falls whereas small gaps were colonized by shade‐tolerant trees (Barden, 1981). In contrast, in subalpine fir forests of coastal British Columbia, Pacific silver fir (*Abies amabilis*) preferentially colonized all gaps regardless of gap size (Lertzman, 1992). However, western hemlock (*Tsuga heterophylla*) was more dominant on stumps in gaps (Lertzman, 1992), suggesting that substrate changes during gap formation could also influence forest dynamics. In temperate coniferous forests of the Pacific Northwest, gap regeneration after tree falls can be quite slow (>25 years, Spies et al., 1990), which results in large areas occupied by canopy gaps (13.1%, Spies et al., 1990). The trees that fall to create these gaps become essential sites of forest regeneration (i.e., nurse logs, Franklin et al., 2002) that cover more area in these forests (up to 25%) than in other forests (<4%; Harmon et al., 1986). Similar to the subalpine fir forests of British Columbia, these nurse logs provide safe germination sites for late succession tree species’, such as shade‐tolerant *Tsuga heterophylla* (Christie & Armesto, 2003; Harmon & Franklin, 1989). Nurse logs are thought to facilitate seedlings through several means, such as by becoming a physical barrier between plants and terrestrial fungal pathogens, limiting competition between seedlings, herbaceous species, and bryophytes on the forest floor, and providing nutrients for potential seedling growth (Franklin et al., 1987; Graham & Cromack, 1982; Harmon, 1986; Harmon & Franklin, 1989). They may also be key to maintaining bryophyte diversity.

Nurse logs are sites for bryophyte colonization and succession. In coniferous forests in Colorado, bryophytes were found to colonize logs after lichen establishment (McCullough, 1948), and in coniferous forests of the Pacific Northwest, epiphytic plants from standing trees were replaced by bryophytes more commonly found on the forest floor (Sharpe, 1956). In the Hoh Rainforest in Washington, bryophyte cover on fallen logs reached approximately 90% in 11–19 years, and succession followed dominance by *Dicranum* spp. and *Hypnum circinale* in early succession, *Rhizomnium* spp. in mid succession, and feather mosses *Kindbergia oregano* and *Hylocomium splendens* in late succession (Harmon, 1989b). Given that late successional bryophytes are tall and dense enough to prevent tree seedling establishment (Harmon, 1986), they may also out‐compete early‐ and mid‐successional bryophyte species. Thus, nurse logs with moderate decay may be a refuge for early‐ and mid‐successional terrestrial bryophytes that have difficulty establishing on the forest floor either due to insufficient light or competition with forest floor plants, such as late successional bryophyte species. Thus, gap dynamics may also be important for the maintenance of bryophyte diversity.

These changes in bryophyte communities may have facilitative or competitive interactions with tree seedlings. In the Hoh rainforest, seedling density followed a humped distribution and was highest when logs were dominated by the mid‐successional bryophytes *Rhizomnium* spp. and lowest when logs were either bare or dominated by late succession bryophytes (Harmon, 1989b). Bare bark is a stressful germination site for tree seedlings because there is little substrate to root into and there is a high risk of drying out; as the log decays, so do the bryophytes, creating a humus substrate that holds nutrients and water into which seedlings can grow (Harmon, 1989b). The largest and fastest growing seedlings surveyed on nurse logs in the Hoh rainforest were those that rooted in masses of dying and decaying bryophytes (Harmon, 1989b). Harmon and Franklin (1989) argue through their controlled experiments that the interaction of bryophytes with seedlings changes from facilitative to competitive once the moss layer exceeds 5 cm in depth. Previous research on bryophyte–vascular plant interactions found that thicker mosses can hinder vascular plants by reducing temperatures and nutrient availability (Gornall et al., 2011; Pearce et al., 2003). However, they could also hinder vascular plant seedlings by reducing light levels if the bryophytes are thick and dense enough. In coniferous forests, removal of forest floor mosses showed positive effects on tree seedlings (Wardle et al., 2008; Zackrisson et al., 1997). Thus, the importance of nurse logs for tree regeneration may be mitigated by bryophyte communities.

Here, we build on the findings of previous studies (Harmon, 1986, 1989b; Harmon & Franklin, 1989) by diving deeper into the role of bryophyte species on tree seedling density. Over the course of three years, we conducted separate studies in northern temperate rainforests on the Olympic peninsula in Washington State (Hoh rainforest) to examine the importance of nurse logs for tree seedlings focusing on the effects of the nurse log bryophyte community, how that could change with nurse log decay class, and how it may differ from the forest floor. We tested the following predictions: (a) tree seedling density would be higher on nurse logs than on the forest floor, as shown previously (Christie & Armesto, 2003; Harmon & Franklin, 1989); (b) bryophyte community composition and the depth of bryophyte mats on nurse logs would influence tree seedling densities (Fukasawa & Ando, 2018; Harmon & Franklin, 1989); and (c) the decay class of the nurse log would influence both bryophyte communities and tree seedling densities. Because bryophytes vary in their growth patterns, we also examined whether particular bryophyte species were more abundant on the forest floor or nurse logs and were associated with high and low tree seedling density as found previously (Harmon, 1989b). We focused on the dominant feather moss, *Hylocomium splendens*, given its abundance in these forests and its impact on tree seedling growth in other studies (Fukasawa & Ando, 2018; Harmon & Franklin, 1989). We tested our hypothesis that the effect of *H. splendens* on seeding growth was due to competition for light by measuring light under and beside this moss. If bryophyte communities on nurse logs change predictably through succession and influence tree seedlings differently at different successional stages, it would further limit the degree of safe regeneration sites in these forests for trees and highlight the essential role of nurse logs and plant–plant interactions in forest dynamics and diversity maintenance.

## 2 MATERIALS AND METHODS

### 2.1 Study site

**FIGURE 1:**
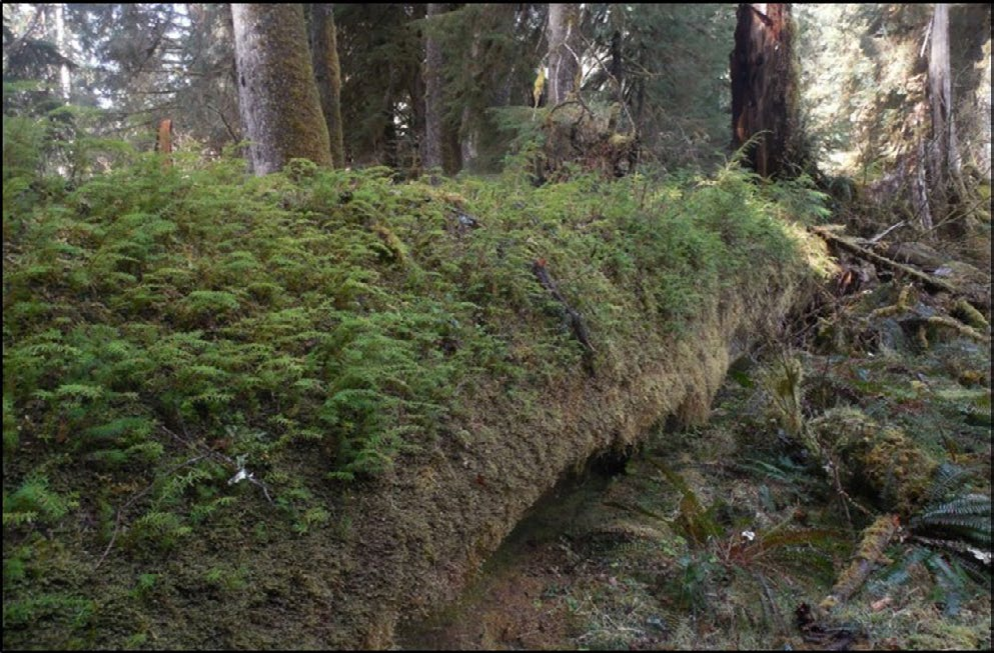
Nurse log of decay class 2 in the Hoh rainforest, WA

Our study was conducted in the Hoh rainforest in the Olympic National Park, a World Heritage Site, and Biosphere reserve in Washington State, USA (47°50′08″N, 123°58′54″W). The climate is mild and extremely wet with an average annual temperature of 10°C (range = −11°C–36°C) and annual rainfall between 3,200 and 3,440 mm (East et al., 2017; Harmon & Franklin, 1989). These forests are characterized by large degrees of vertical and horizontal heterogeneity formed by large and tall trees (>80 m) that are dominated by *Picea sitchensis* and *Tsuga heterophylla*, an abundance of large nurse logs (>1 m diameter, Figure 1), a luxurious cover of bryophytes and herbs on the forest floor, and an abundance of epiphytic bryophytes (Franklin et al., 2002; Harmon & Franklin, 1989).

### 2.2 Survey methods

We conducted this study over the course of three summers (2016–2018). We counted *Tsuga heterophylla* and *Picea sitchensis* seedlings in 166 randomly placed 1 m × 1 m plots on the forest floor (*n* = 52) and 1 m × nurse log diameter on nurse logs (defined as >30 cm diameter and at least 2 m in length; nurse log diameter ranged from 30 to 104 cm; *n* = 114). For a subset of the nurse log plots (*n* = 86) and forest floor plots (*n* = 12), bryophyte composition was surveyed using the point‐intercept method whereby each bryophyte species found under each of 100 randomly placed dots on a transparent sheet was noted. Percent canopy cover was measured as a potential confounding factor using a densiometer placed in the center of each plot. Bryophyte depth was measured (cm) using a caliper in the center of each plot. This single value of depth was compared with the average of 10 randomly placed depth measurements in a subset of the plots (45) and was found not to be statistically significant (*t* test, *p* < 0.05). Thus, we used the single depth value in our analyses. Because Harmon and Franklin (1989) found that seedlings that receive less than 0.6% of light (<12.5 µmol m^−2^ s^−1^) would not be able to survive, and seedling survival was reduced with high moss biomass (an indicator of moss depth), we also measured light irradiance with a photometer in 50 pairs under and beside thick bryophyte mats focusing on the predominant moss in the forest, *Hylocomium splendens*. Decay class of each nurse log for each plot was measured using the kick test, which included three levels of decay classes: 1 = bark still intact upon contact, 2 = some to various debris falls upon contact, and 3 = foot completely in log, several pieces fragmented off of the log (modified from Christy & Mack, 1984; Sollins et al., 1987, and Fogel, Ogawa, and Trappe, *unpublished report*). These classes correlate with the age of the nurse log so we used them as an indicator of nurse log age (Harmon, 1989b).

### 2.3 Statistical analysis

To test our first prediction that tree seedling density (number of seedlings per m^2^) was higher overall on nurse logs than on the forest floor, we used a Wilcoxon rank test because the data did not meet parametric model assumptions of normality and homogeneity of variance across groups. We used seedling density per area of the plot because area of the plot varied with nurse log diameter. We then examined whether substrate type including forest floor and all three decay classes of nurse logs influenced tree seedling density, bryophyte depth, percent canopy cover, and percent cover of the three most abundant bryophyte species (*Rhizomnium glabrescens*, *Hylocomium splendens*, *Antitrichia curtipendula*) using one‐way ANOVA or Kruskal–Wallis tests if parametric model assumptions could not be met. Bryophyte depth was missing from one plot and percent cover of *H*. *splendens* had to be square‐root transformed to meet parametric model assumptions.

To examine the influence of substrate (forest floor and nurse logs characterized by decay class) on bryophyte community composition, we conducted a nonmetric multi‐dimensional scaling ordination (NMDS) using the metaMDS function in the vegan package in R (Oksanen et al., 2010). We used a Bray–Curtis distance matrix on bryophyte percent cover in each plot. Only plots that had at least two bryophyte species were included (*n* = 98). We used ellipses to denote variances in bryophyte species composition among substrate types. To test our second prediction that bryophyte composition and depth would influence tree seedlings, we fit bryophyte depth (BD), tree seedling density (seedling), and percent canopy cover (CC) as environmental vectors to the NMDS ordination using the envfit function in the vegan package in R with 1,000 permutations (Oksanen et al., 2010). To test our third prediction that decay class would influence bryophyte communities, we tested for significant differences in species composition among substrate types using the adonis function in the vegan package, which is essentially a multivariate analysis of variance. We then used the pairwiseAdonis function in the vegan package to compute all pairwise comparisons among substrate types with adjusted *p*‐values (Martinez Arbizu, 2020).

To determine the factors associated with seedling density (predictions 2 and 3), we used a generalized linear model (GLM) with a Poisson error distribution. Explanatory variables included substrate type (nurse log or forest floor), decay class of nurse log, bryophyte depth, % canopy cover, and bryophyte species with a total percent cover of >300 across all plots and that were found in at least 10 plots (Table 1). The bryophyte species included as predictors were *Hylocomium splendens*, *Rhizomnium glabrescens*, *Antitrichia curtipendula*, *Rhytidiadelphus loreus*, *Sphagnum girgensohnii*, and *Kindbergia praelonga* (Table 1). We used the dredge function in the Mu‐MIn package to determine the model with the lowest Akaike information criterion (AIC) value (Barton, 2009). We calculated Spearman rank correlation coefficients for all pairs of explanatory variables using the rcorr function in the Hmisc package in R (Harrell & with contributions from Charles Dupont and many others, 2020). We included forest floor as decay class 4 in our model. We also calculated variance inflation factors (vif) to test for collinearity among our predictor variables using the vif function in the car package; any vif <5 was deemed not correlated.

**TABLE 1:** Variables used as predictors of tree seedling density in the generalized linear model

Variable	Data type	Min, Mean, Max
Substrate (nurse log or forest floor)	Categorical	n/a
Decay class of nurse log	Rank	1, –, 3
Moss depth	Continuous, cm	0, 6.1, 20.6
Canopy cover above plot	Continuous, %	74.9, 93.3, 99.8
*Hylocomium splendens* cover	Continuous, %	0, 23.5, 98
*Rhizomnium glabrescens* cover	Continuous, %	0, 3.5, 41
*Antitrichia curtipendula* cover	Continuous, %	0, 9.5, 88
*Rhytidiadelphus loreus* cover	Continuous, %	0, 3.2, 94
*Sphagnum girgensohnii* cover	Continuous, %	0, 4.1, 94
*Kindbergia praelonga* cover	Continuous, %	0, 7.2, 65

To test the light levels under and beside *Hylocomium splendens* mats, we used a paired *t* test. We used R v3.6.0 for all analyses (R Core Team & R Development Core Team, 2020).

## 3 RESULTS

### 3.1 Substrate type

In a total of 166 plots, tree seedlings were found in 50% of our plots. Of those 166 plots, 52 were on the forest floor and 114 were on nurse logs (Figure 1); 36% of the forest floor plots had tree seedlings whereas 57% of the nurse log plots had tree seedlings. *Tsuga heterophylla* was the dominant tree species found as seedlings (87% of all seedlings found). On nurse logs, we found a total of 18 different nonvascular species. Of these 18, there were 16 mosses, one liverwort, and one lichen (Table 2). We found only six different bryophyte species on the forest floor (Table 2).

**TABLE 2:** List of nonvascular species found on nurse logs of three different decay classes and the forest floor (*n* = 93) in the Hoh rainforest

Family	Species	Species code	Nurse log 1	Nurse log 2	Nurse log 3	Forest floor	Total
Leucodontaceae	*Antitrichia curtipendula* (Hedw.) Brid. var. curtipendula	AntCur	309	450	186	34	979
Climaciaceae	*Climacium dendroides* (Hedw.) F. Weber & D. Mohr	CliDen	18	0	0	0	18
Dicranaceae	*Dicranum fuscescens* Turner	DicFus	16	78	70	0	164
Brachytheciaceae	*Homalothecium fulgescens* (Mitt. ex C. Müll.) Lawt.	HomFul	20	7	0	0	27
Hylocomiaceae	*Hylocomium splendens* (Hedw.) Schimp.	HylSpl	92	635	615	742	2,084
Hypnaceae	*Hypnum subimponens* Lesq.	HypSub	1	13	0	0	14
Brachytheciaceae	*Isothecium myosuroides* Brid.	IsoMyo	55	2	49	0	106
Brachytheciaceae	*Kindbergia oregana* (Sullivant) Ochyra	KinOre	121	14	95	0	230
Brachytheciaceae	*Kindbergia praelonga* (Hedwig) Ochyra, Lindbergia	KinPra	285	163	123	56	627
Mniaceae	*Leucolepis acanthoneuron* (Schwägr.) Lindb.	LeuAca	5	0	0	0	5
Lobariaceae	*Lobaria oregana* (Tuck.) Müll. Arg.	LobOre	2	1	1	0	4
Sphagnaceae	*Sphagnum girgensohnii* Russow	SphGla	0	79	5	59	143
Plagiotheciaceae	*Plagiothecium undulatum* (Hedw.) Schimp.	PlaUnd	22	6	148	0	176
Mniaceae	*Rhizomnium glabrescens* (Kindb.) T. Kop.	RhiGla	179	76	19	0	274
Mniaceae	*Rhizomnium magnifolium* (Horikawa) T. J. Koponen	RhiMag	47	66	1	0	114
Hylocomiaceae	*Rhytidiadelphus loreus* (Hedw.) Warnst.	RhyLor	19	21	37	10	87
Hylocomiaceae	*Rhytidiadelphus triquetrus* (Hedw.) Warnst.	RhyTri	27	25	0	0	52
Scapaniaceae	*Scapania bolanderi* Austin	ScaBol	24	29	10	1	64
	Sample size		31	30	25	12	

**FIGURE 2:**
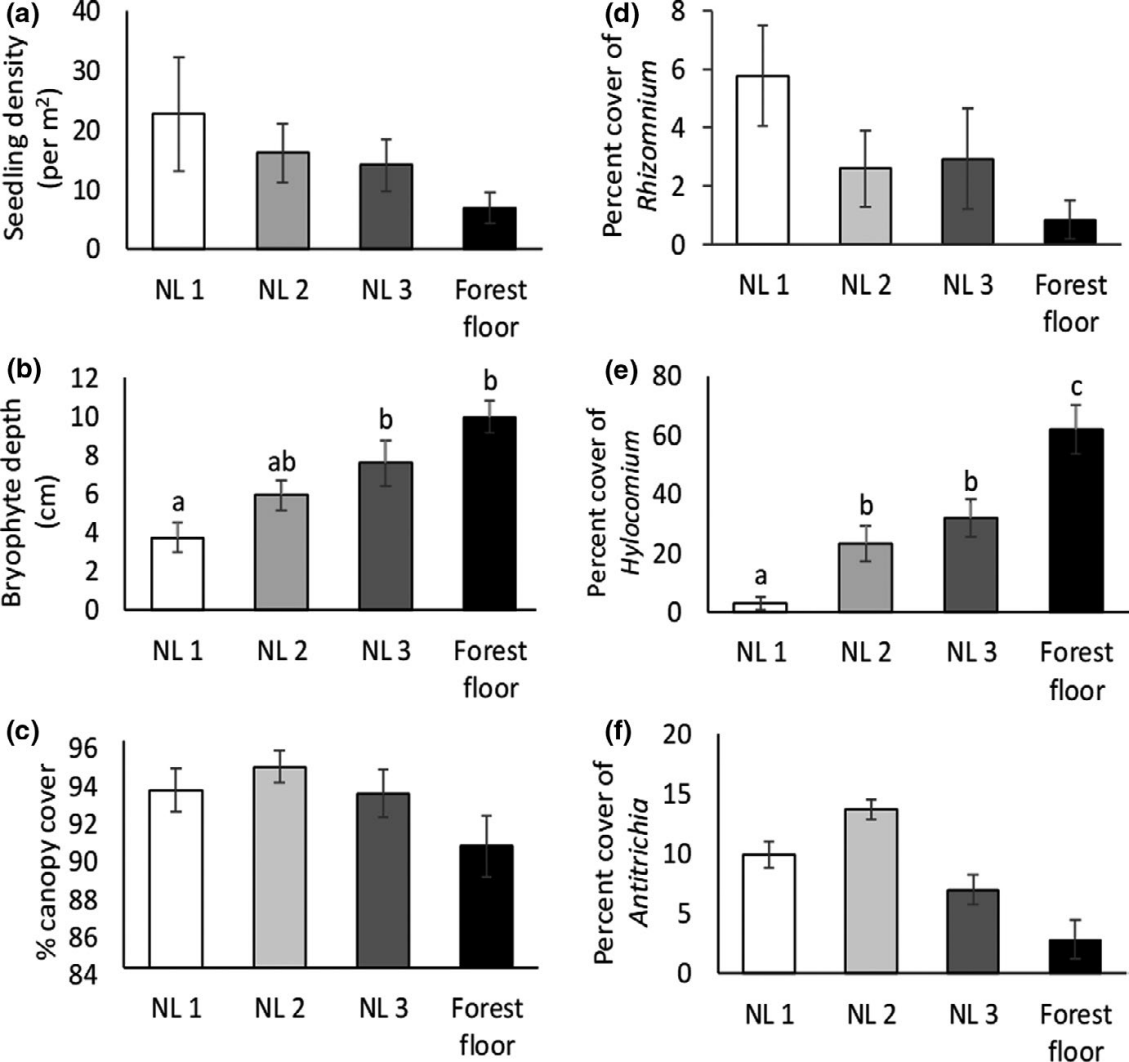
Average (± *SE*) tree seedling density of *Tsuga heterophylla* and *Picea sitchensis* (a), bryophyte depth (b), percent canopy cover (c), percent cover of *Rhizomnium glabrescens* (d), percent cover of *Hylocomium splendens* (e), and percent cover of *Antitrichia curtipendula* (f) on the forest floor (*n* = 12) and each decay class of nurse logs (NL 1 = nurse log decay class 1, *n* = 31; NL 2 = nurse log decay class 2, *n* = 30; NL 3 = nurse log decay class 3, *n* = 25) in a northern temperate rainforest, Olympic peninsula, WA. Bryophyte depth was missing from one plot. Substrate type significantly influenced bryophyte depth (*F*_3,93_ = 6.6, *p* < 0.001). Substrate type significantly influenced percent cover of *H. splendens* (*F*_3,94_ = 17.8, *p* < 0.001)

Tree seedling density was significantly influenced by substrate type, as average tree seedling density on nurse logs (20.1 ± 3.6) was 4.6× greater than on the forest floor (4.5 ± 1.8; *W* = 2,027.5, *p* < 0.001). Substrate type significantly influenced moss depth (*F*_3,93_ = 6.6, *p* < 0.001, Figure 2b), and the percent cover of *H. splendens* (*F*_3,94_ = 17.8, *p* < 0.001, Figure 2e). No other factor was significantly influenced by substrate type (Figure 2).

### 3.2 Bryophyte composition and depth

**FIGURE 3:**
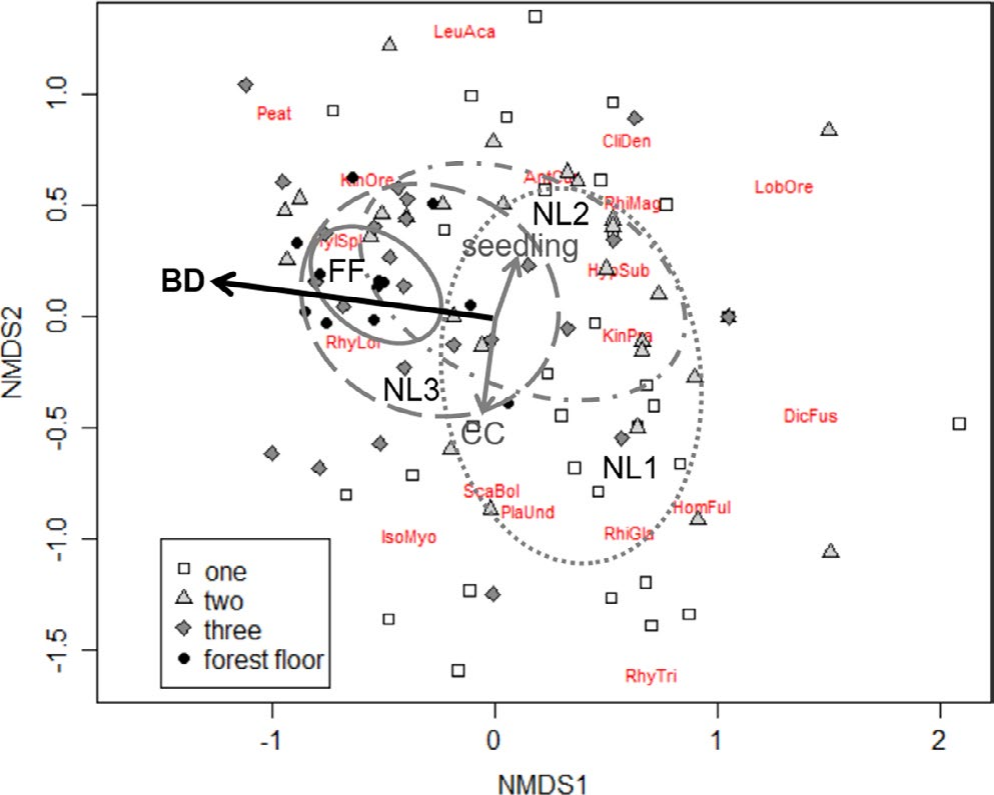
Nonmetric multi‐dimensional (NMDS) ordination of bryophyte community composition on nurse logs (*n* = 98) of varying decay stages and the forest floor using a Bray–Curtis distance matrix on percent cover. Two‐dimensional stress = 0.16 with 1,000 permutations. The ellipses show the covariance matrix centered on the mean of each nurse log decay stage and the forest floor (solid black line). Nurse logs were classified into three decay stages (modified from Christy & Mack, 1984): NL1 = bark still intact and can hold weight when stepped on (light gray dotted line), NL2 = some to various debris falls upon contact (gray dashed dot line), and NL3 = foot completely in log, several pieces fragmented off of the log (dark gray dashed line). Forest floor = FF (solid gray line). Species codes are from Table 2. Environmental variables that significantly correlated with NMDS axes are shown in black, heavy arrows and those that did not significantly correlate with axes are shown in gray. Bryophyte depth (BD) correlated significantly with the axes (*r* ^2^ = 0.47, *p* < 0.001) but not canopy cover (CC, *r* ^2^ = 0.06, *p* = 0.08) or tree seedling density (seedling, *r* ^2^ = 0.02, *p* = 0.4). Species codes are in red and are the first three letters of the genus and the first three letters of the species (found in Table 2)

Bryophyte composition varied significantly with substrate type (adonis, *F*_3,97_ = 6.8, *p* = 0.001, Figure 3). Decay class 1 was significantly different from decay class 3 (*p* = 0.006) and the forest floor (*p* = 0.006); there were no other significant differences in bryophyte composition among substrate types (*p* > 0.01, Figure 3). These differences were driven by bryophyte species that create thick mats (>4 cm), such as *Hylocomium splendens* (HylSpl), which were associated with the forest floor and later decay classes of nurse logs (class two and three), whereas bryophyte species that do not create thick mats were associated with early decay classes of nurse logs (one), such as *Rhizomnium glabrescens* (RhiGla) and *Scapania bolanderi* (ScaBol; Figure 3). Bryophyte depth was the only factor that had a significant correlation with bryophyte community composition (*r* ^2^ = 0.47, *p* <  0.001). While seedling density was higher on nurse logs that were in the early decay class, it did not significantly correlate with bryophyte composition (*r* ^2^ = 0.02, *p* = 0.4). Canopy cover did not significantly correlate with the NMDS ordination but showed a trend toward decay class 2 (*r* ^2^ = 0.06, *p* = 0.06).

The GLM model that best predicted tree seedling density included substrate type, percent canopy cover as a positive factor, and percent cover of *Hylocomium splendens* as a negative factor (Table 3). Interestingly, moss depth was a negative factor and percent cover of *Rhizomnium glabrescens* was a positive factor, so their exclusion from the model may be due to their correlative relationships with the percent cover of *H. splendens*.

**TABLE 3:** Results of the generalized linear model (GLM) analysis showing the predictors of tree seedling density from the best model

Factor	Estimate	*SE*
Canopy cover	0.019***	0.005
*Hylocomium* cover	−0.009***	0.001
Substrate	0.315*	0.136
AIC of the best model	4,269.6	

* *p* < 0.05

*** *p* < 0.0001.

### 3.3 Decay class

Decay class was significantly positively associated with moss depth and the percent cover of *Hylocomium splendens* (Table 4). Moss depth was positively correlated with the percent cover of *H. splendens* and *Rhytidiadelphus loreus* and negatively correlated with the percent cover of *Rhizomnium glabrescens* (Table 4). Percent cover of *H. splendens* was negatively correlated with the percent cover of *Kindbergia praelonga*. Finally, the percent canopy cover was positively correlated with the percent cover of *Antitrichia curtipendula* (Table 4).

**TABLE 4:** Spearman rank correlation coefficients between explanatory variables for the generalized linear model for seedling density. Bryophytes are listed by genus only (see Table 2 for species names)

	Decay class	Bryophyte depth	*Hylocomium*	Canopy cover	*Rhizomnium*	*Antitrichia*	*Rhytidiadelphus*	*Sphagnum*
Decay class								
Bryophyte depth	**0.41** ***							
*Hylocomium*	**0.56** ***	**0.58** ***						
Canopy cover	−0.16	−0.1	−0.02					
*Rhizomnium*	−0.18	**−0.25** *	−0.17	−0.13				
*Antitrichia*	−0.13	0.05	−0.17	**0.32** **	−0.19			
*Rhytidiadelphus*	0.05	**0.4** ***	0.04	−0.16	0.03	−0.1		
*Sphagnum*	0.12	0.19	−0.01	−0.05	−0.12	−0.12	−0.03	
*Kindbergia*	−0.13	−0.21	**−0.24** *	−0.14	0.11	−0.08	−0.11	−0.15

Note

Values in bold indicate a significant correlation between variables.

*** *p* < 0.001

** *p* < 0.01

* *p* < 0.05.

**FIGURE 4:**
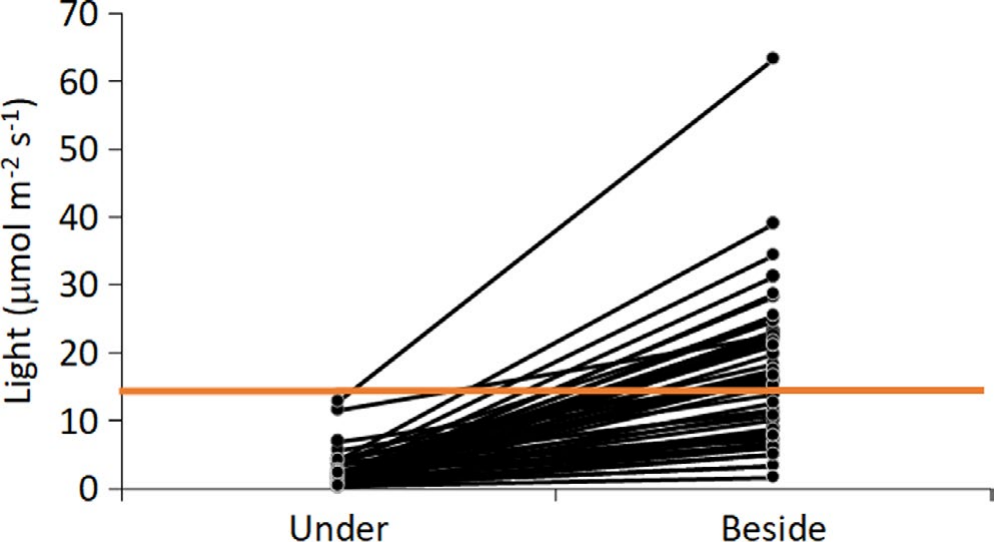
Light irradiance (µmol m^−2^ s^−1^) under and beside 50 pairs of *Hylocomium splendens* individuals in the Hoh rainforest. Red line denotes the level under which tree seedling survival declines (<12.5 µmol m^−2^ s^−1^, Harmon & Franklin, 1989)

Light levels were significantly lower under *Hylocomium splendens* than beside it (paired *t* test, *t* = −10.3, *df* = 49, *p* < 0.001, Figure 4).

## 4 DISCUSSION

**FIGURE 5:**
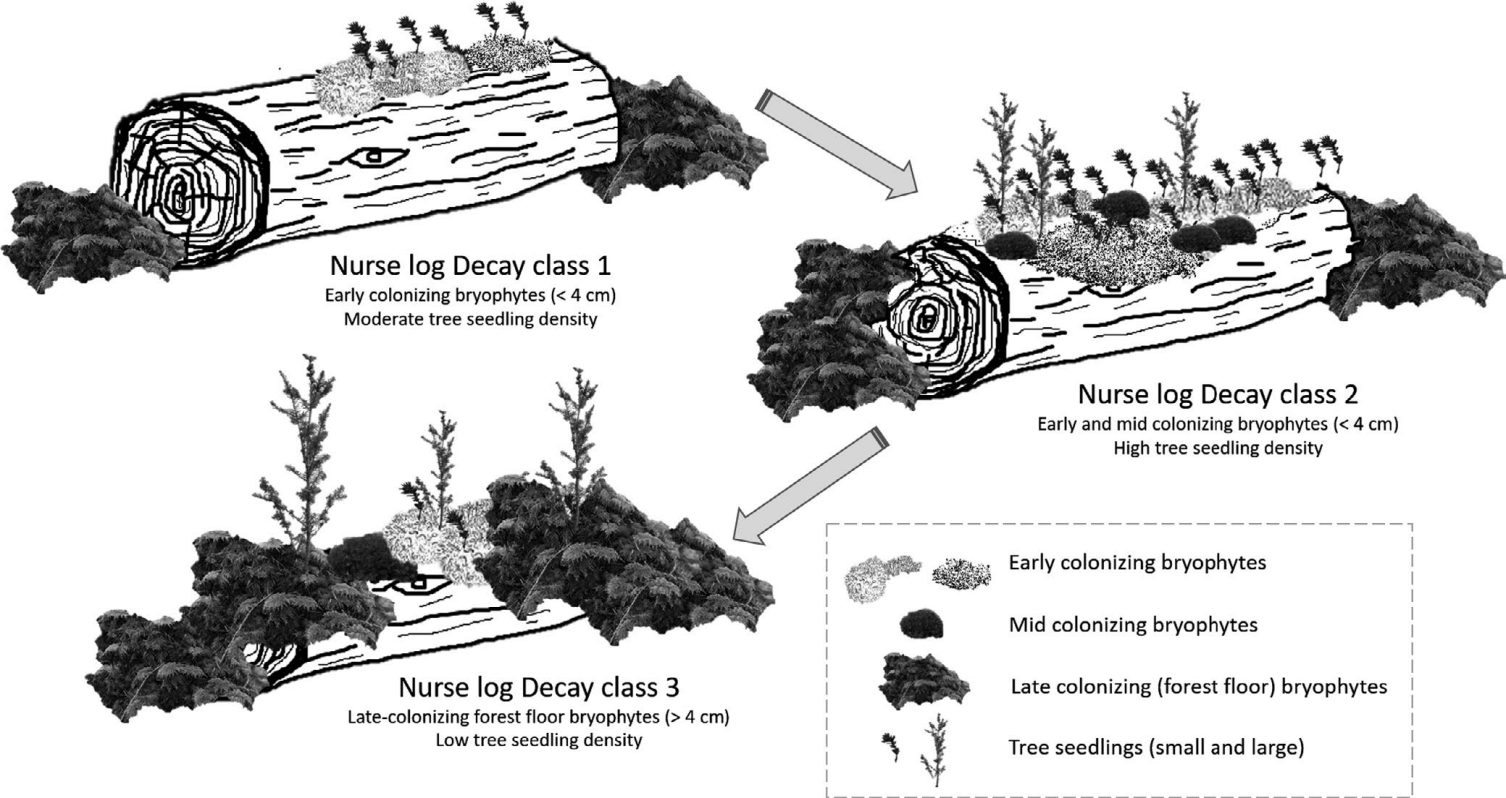
Graphical depiction of how bryophyte communities and tree seedlings change through nurse log decay in this northern temperate rainforest. Bryophyte depth increases through decay as communities shift from short, early‐colonizing species (<4 cm) to tall, late colonizing species (>4 cm) that also dominate on the forest floor. Early colonizing bryophytes facilitate tree seedlings while late colonizing bryophytes competitively exclude seedlings

Our study found that tree seedlings were largely affected by bryophyte species identity and the thickness of the bryophyte cover. We found support for our first prediction as overall tree seedlings were much more abundant on nurse logs than on the forest floor as found previously (Christy & Mack, 1984; Harmon & Franklin, 1989). However, not all nurse logs were created equal; nurse logs at early decay classes supported many tree seedlings and small bryophyte species, such as *Rhizomnium glabrescens* and *Scapania bolanderi*, while later decay classes had few tree seedlings and bryophyte species with greater depths, such as *Hylocomium splendens* (Figure 5). Moreover, the percent cover of *H*. *splendens* was negatively associated with tree seedling density and positively associated with bryophyte depth. Thus, we found support for our second and third predictions. Our findings are in accordance with Harmon (1989a, 1989b) who found seedling densities highest when nurse logs were dominated by *Rhizomnium* spp. and lowest when dominated by *H. splendens*. Our findings are also supported by another study conducted in old‐growth subalpine spruce forests in Japan where tree seedlings on nurse logs were found to be positively associated with a small liverwort, *Scapania bolanderi*, and negatively associated with *H. splendens* (Fukasawa & Ando, 2018). As in our study, Fukasawa and Ando (2018) also found that bryophyte community composition shifted from dominance by *S. bolanderi* to *H. splendens* as nurse logs decayed. Thus, bryophyte succession on nurse logs influenced tree seedlings differently depending on the stage of succession (Figure 5).

As nurse logs decayed, the relationship between bryophyte communities and tree seedlings changed from facilitation to competition. We found support for this in our study as the pattern of seedling density mirrored that of the percent cover of *Rhizomnium glabrescens* (Figure 2a,d) while was opposite that of the percent cover of *Hylocomium splendens* (Figure 2a,e), and *H. splendens* came out in the model as the only negative driver of seedling density. These patterns are consistent with other studies. In subalpine forests of Japan, *Tsuga* seedling survival was restricted to nurse logs covered with small bryophyte species (Nakamura, 1992), and in the Hoh rainforest, seedling establishment was highest on logs with thin mosses as seed retention on bare logs was low, and thick mosses excluded seedlings (Harmon & Franklin, 1989). While our study and others found correlative support of these relationships, findings from manipulative field experiments provide the strongest evidence. In old‐growth forests in western Oregon, for example, seedling emergence was higher on nurse logs of decay class 3 (our class 2) when bryophytes were present than when they were removed (Christy & Mack, 1984), and in a field experiment in the Hoh rainforest testing the influence of moss depth on seedling density, seedling density of *Tsuga* was higher in a moss mat of 3.7 cm than in 1.4 cm or 7.8 cm (Harmon & Franklin, 1989). The positive effect of bryophytes on tree seedlings could be due to protection from herbivores, increasing moisture or nutrient availability, protecting seeds from being blown off by the wind, or providing a humus substrate into which seedling roots can grow (Graham & Cromack Jr., 1982; Harmon & Franklin, 1989; Nakamura, 1992; Sand‐Jensen & Hammer, 2012). Facilitation of tree seedlings may also be linked to bryophyte richness as greater species richness of bryophytes was found to have increased moisture absorption and retention (Rixen & Mulder, 2005). On average, bryophyte species richness in our study was highest on nurse log decay class 1 and 2, which may increase seedling germination and survival. Many studies have found competitive interactions between bryophytes and tree seedlings (Christy & Mack, 1984; Fukasawa & Ando, 2018; Harmon & Franklin, 1989; Nakamura, 1992). The negative effect of thick bryophytes on tree seedlings could be due to preventing seeds from reaching the humus or soil layer, which can inhibit germination (Iijima & Shibuya, 2010), the reduction in light levels below the threshold needed for tree seedling germination and survival as found in our study and others (Harmon, 1986; Iijima & Shibuya, 2010), or reducing nitrogen availability to vascular plants as found in the boreal forest (Gornall et al., 2011). The “window of time” for tree seedling establishment on logs of a particular stage of decomposition seems to be driven by bryophyte succession on logs (Fukasawa & Ando, 2018), suggests that the available substrate for successful seedling regeneration is even more limited than the area covered by nurse logs would suggest.

The benefits of nurse logs to tree seedlings could also be due to a release from competition with forest floor plants. In western Oregon old‐growth forests dominated by *Pseudotsuga menziesii* and *Tsuga heterophylla*, 98% of the seedlings in the plot were on nurse logs despite the fact that nurse logs only covered 6% of the plot, and *T. heterophylla* emergence was higher on mineral soil when litter, bryophytes (such as *Hylocomium splendens*) and herbaceous plants were removed than in the forest floor control where nothing was removed (Christy & Mack, 1984). In the Hoh rainforest where our study took place, tree seedling survival was significantly higher on the forest floor when bryophytes and vascular plants were removed (Harmon & Franklin, 1989). Our findings corroborate this, as we found lower seedling densities on the forest floor where *H. splendens* was in high abundance. Our finding that *H. splendens* reduces light levels below the threshold for seedling survival (<12.5 µmol m^−2^ s^−1^) suggests that the mechanism by which *H. splendens* out‐competes tree seedlings is through light competition as suggested previously (Harmon & Franklin, 1989). Christy and Mack (1984) also suggest that nurse logs enable seedlings to avoid being covered by litter that dominates the forest floor in western Oregon forests. Interestingly, the small tree seedlings (<15 cm) that were on the forest floor in our study were on areas covered by litter where bryophytes were not abundant. The tree seedlings we found on the forest floor or mounds of very decayed nurse logs that were covered in *H. splendens* were quite tall (>15 cm, Figure 5), which suggests that seedlings that establish on nurse logs prior to *H. splendens* could grow tall enough to surpass the height of the moss. This could add bryophyte cover as a contributor to the decrease in tree seedling survival after the first 1–2 years of growth (Christy & Mack, 1984; Friesner & Potzger, 1944; Haig et al., 1941). While litter may negatively impact tree seedlings, manipulative field experiments with long‐term seedling survival data are further required to disentangle the relative influence of bryophytes and litter on tree seedlings both on the forest floor and on nurse logs.

On nurse logs, what drives the initial colonization and subsequent turnover in species composition is largely unknown. Rodents and slugs have been found to disperse lichen and bryophyte propagules (Asplund et al., 2010; Barbé et al., 2016; Kimmerer, 1994; Kimmerer & Young, 1995), and mammals use fallen logs as trails through the forest, so it could be a combination of differences in propagule dispersal ability, germination requirements, and biotic interactions that determine these changes. Alternatively, different decomposer fungi could influence bryophyte community structure on nurse logs as found in alpine forests in Japan (Ando et al., 2017; Fukasawa et al., 2019). Thus, the influence of bryophytes on tree seedlings could be mediated by other biotic interactions through a trophic cascade. The change in bryophyte communities could also be influenced by the vascular plants. A study examining the interactions of vascular plants and *Sphagnum* mosses in peat bogs found that *Sphagnum* growth was improved by a low density of vascular plants but less so by a high density of vascular plants (Pouliot et al., 2011). Interactions, such as facilitation, among bryophyte species themselves have also been found to drive succession (Fenton & Bergeron, 2006). Regardless, we did find that nurse logs are essential not only for the forest regeneration but also for the maintenance of bryophyte diversity in late stage forests. We found 17 bryophyte species on nurse logs and only six on the forest floor, and there were significant differences in species composition between our nurse log decay class 1 and the forest floor. Without gap‐phase dynamics driven by large tree falls, these early nurse log bryophyte species would eventually be competitively excluded by the late stage bryophytes.

Our results support the predictions of the Stress Gradient Hypothesis that plant–plant interactions are complex and can vary from facilitative to competitive depending on the severity of the external environment (Bertness & Callaway, 1994). For tree seedlings, some level of bryophyte cover on nurse logs seems to be needed to facilitate germination and seedling establishment, while too much bryophyte cover inhibits germination and seedling survival. *Hylocomium splendens*, in particular, has been found to consistently exclude seedlings (Fukasawa & Ando, 2018; Harmon & Franklin, 1989). This is likely due to *H. splenden's* distinctive “stairstep” architecture, which lends it unusual height for a bryophyte. The average life span of *Hylocomium splendens* is 8 years (Binkley & Graham, 1981), and it dominates nurse logs that are >30 years old (Harmon, 1989a). Forest floor bryophytes may have an almost unlimited life span given their continued upward growth (Fenton & Bergeron, 2006). Thus, unless there is a disturbance that disrupts the dominance by these forest floor bryophytes, the regeneration of the dominant trees will be limited to the small window of opportunity found in early successional stages of bryophytes communities on nurse logs, which is less than 30 years. Interestingly, above the alpine tree line in subarctic tundra in Sweden, spruce tree establishment was improved when planted in bryophyte mats of *Hylocomium splendens* (Lett et al., 2017), and vascular plants were found to facilitate *Sphagnum* mosses in peat bogs in Canada (Pouliot et al., 2011) further supporting that species interactions fall along a spectrum depending on site conditions. Bryophytes, though often overlooked in forested ecosystems, could be an important driver of plant community composition and dynamics.

## CONFLICT OF INTEREST

Authors declare no conflict of interest.

## AUTHOR CONTRIBUTIONS

**Carrie L. Woods:** Conceptualization (lead); Data curation (lead); Formal analysis (lead); Funding acquisition (equal); Investigation (equal); Methodology (lead); Project administration (lead); Resources (lead); Supervision (lead); Validation (lead); Visualization (lead); Writing‐original draft (lead); Writing‐review & editing (equal). **Katy Maleta:** Conceptualization (equal); Formal analysis (equal); Funding acquisition (equal); Investigation (equal); Methodology (supporting); Visualization (supporting); Writing‐review & editing (equal). **Kimmy Ortmann:** Conceptualization (equal); Formal analysis (supporting); Funding acquisition (equal); Investigation (supporting); Methodology (supporting); Writing‐review & editing (equal).

### OPEN RESEARCH BADGES

This article has earned an Open Data Badge for making publicly available the digitally‐shareable data necessary to reproduce the reported results. The data is available at https://doi.org/10.5061/dryad.5x69p8d34.

## Data Availability

A list of tree seedling density, *Tsuga heterophylla* seedling density, percent canopy cover, bryophyte depth, and the percent cover of the most abundant bryophyte species in each plot are deposited in the Dryad depository: DOI https://doi.org/10.5061/dryad.5x69p8d34.
